# Intergenerational and intrafamilial phenotypic variability in 22q11.2 Deletion syndrome subjects

**DOI:** 10.1186/1471-2350-15-1

**Published:** 2014-01-02

**Authors:** Emilia Cirillo, Giuliana Giardino, Vera Gallo, Pamela Puliafito, Chiara Azzari, Rosa Bacchetta, Fabio Cardinale, Maria Pia Cicalese, Rita Consolini, Silvana Martino, Baldassarre Martire, Cristina Molinatto, Alessandro Plebani, Gioacchino Scarano, Annarosa Soresina, Caterina Cancrini, Paolo Rossi, Maria Cristina Digilio, Claudio Pignata

**Affiliations:** 1 Department of Translational Medicine, “Federico II” University, Naples, Italy; 2 Department of Pediatrics, (DPUO), University of Rome Tor Vergata, Rome, Italy; 3 Department of Pediatrics, Anna Meyer Children’s University Hospital, Florence, Italy; 4 San Raffaele Telethon Institute for Gene Therapy (HSR-TIGET), Milan; Pediatric ImmunoHematology IRCCS San Raffaele Hospital, Milan, Italy; 5 Department of Pediatrics, Giovanni XXIII Pediatric Hospital, Bari, Italy; 6 Pediatric ImmunoHematology IRCCS, San Raffaele Hospital, Milan, Italy; 7 Department of Internal and Experimental Medicine, University of Pisa, Pisa, Italy; 8 Department of Pediatrics, University of Turin, Turin, Italy; 9 Department of Biomedicine and Evolutive Aging, University of Bari, Bari, Italy; 10 A. Nocivelli Institute for Molecular Medicine, Pediatric Clinic, University of Brescia, Brescia, Italy; 11 Medical Genetics Unit, General Hospital G. Rummo, Benevento, Italy; 12 Medical Genetics, Bambino Gesù Pediatric Hospital, IRCCS, Rome, Italy; 13 Department of Translational Medical Sciences, Unit of Pediatric Immunology, “Federico II” University, via S. Pansini, 5-80131 Naples, Italy

**Keywords:** 22q11.2 deletion syndrome, DiGeorge syndrome, Immunodeficiency, Phenotypic variability

## Abstract

**Background:**

22q11.2 deletion syndrome (22q11.2DS) is a common microdeletion syndrome, which occurs in approximately 1:4000 births. Familial autosomal dominant recurrence of the syndrome is detected in about 8-28% of the cases. Aim of this study is to evaluate the intergenerational and intrafamilial phenotypic variability in a cohort of familial cases carrying a 22q11.2 deletion.

**Methods:**

Thirty-two 22q11.2DS subjects among 26 families were enrolled.

**Results:**

Second generation subjects showed a significantly higher number of features than their transmitting parents (212 vs 129, *P* = 0.0015). Congenital heart defect, calcium-phosphorus metabolism abnormalities, developmental and speech delay were more represented in the second generation (*P* < 0.05). Ocular disorders were more frequent in the parent group. No significant difference was observed for the other clinical variables. Intrafamilial phenotypic heterogeneity was identified in the pedigrees. In 23/32 families, a higher number of features were found in individuals from the second generation and a more severe phenotype was observed in almost all of them, indicating the worsening of the phenotype over generations. Both genetic and epigenetic mechanisms may be involved in the phenotypic variability.

**Conclusions:**

Second generation subjects showed a more complex phenotype in comparison to those from the first generation. Both ascertainment bias related to patient selection or to the low rate of reproductive fitness of adults with a more severe phenotype, and several not well defined molecular mechanism, could explain intergenerational and intrafamilial phenotypic variability in this syndrome.

## Background

Chromosome 22q11.2 deletion syndrome (22q11.2DS), also known as DiGeorge or velocardiofacial syndrome (OMIM#188400), occurs in approximately 1:4000 live births [1,2]. Major clinical features include facial anomalies, conotruncal cardiac defects, palatal anomalies, neonatal hypocalcaemia, mild to moderate immune deficiency related to thymic a/hypoplasia [3-5], developmental and speech delay [6]. Ocular, renal and skeletal anomalies may also be found. However, the syndrome has a very wide spectrum of phenotypic features [7,8]. Evidence highlighted that subjects carrying the deletion may have only mild phenotypes [9,10]. Psychiatric or autoimmune disorders [11] can be the features leading to the diagnosis in adolescents and adults, and, in particular, among adults, they may be the unique clinical feature [12,13]. The identification of subjects with attenuated phenotypes is leading to the understanding that the syndrome is more frequent than previously thought and to focus on novel atypical presentations. A wide clinical variability has also been reported even within the same family [9,10,14,15] and a phenotypic discordance has been described among monozygotic twins [16,17]. Genetic modifiers, chance association or environmental interactions have been proposed to explain the intrafamilial variability. Somatic mosaicism or post zygotic second hit have also been hypothesized as potential mechanisms underlying such phenotypic discordance, even though, to date, no definitive explanation is available.

The deletion results from non allelic homologous recombination, occurring during meiosis and mediated by low-copy repeats (LCR) on chromosome 22 [18-20]. Most patients have a deletion of the same 3 Mb region on 22q11.2, including about 30 genes, whereas in 8% of the cases a smaller deletion of 1.5 Mb, which contains 24 genes, is found. So far, no correlation between the severity of the phenotype and the different size of the deletion has been documented [21]. Both deletions include the *TBX1* gene, a member of the T-box family genes. Mice, haploinsufficient for *TBX1,* share several features with humans carrying the homologous deletion, and, in particular, structural cardiac anomalies [22]. Interestingly, both gain or loss of function mutations in *TBX1* have been reported in human subjects exhibiting a DiGeorge-like phenotype [23].

In most cases, the deletion is a sporadic event, while in 8-28% of the cases the syndrome is inherited in an autosomal dominant fashion [8,10,24-26].

Several studies have analyzed the phenotypic variability of the syndrome, but an extensive and conclusive intergenerational and intrafamilial comparison has not yet been reported.

The aim of this study is to perform an intergenerational and intrafamilial comparison of the clinical phenotype in a cohort of patients affected with inherited chromosome 22q11.2DS.

## Methods

### Patients

Thirty-two subjects (18 females) affected with familial 22q11.2DS from 26 families, were enrolled into the study. The study and data collection, approved by the local Ethics Committee for Biomedical activities “Carlo Romano”, have been performed upon informed consent and in compliance with the Helsinki Declaration (http://www.wma.net/en/30publications/10policies/b3/index.html). Within the group, 17 subjects were from the Italian Network for Primary Immunodeficiencies (IPINET) Registry, followed at 8 Italian Centers, and 15 were referred to two Genetic Units. In 4 families, 2 affected siblings were diagnosed and in one further family, 3 subjects were identified. Mean age +/− SD was 10.4 +/− 7.23 years (range 4 months-31 years). The parent carrying the deletion was the mother in 17 cases (65%) and the father in 9 (35%). Mean age +/− SD of carrier parents was 39.8 +/− 7.9 years (range 21–58 years). We found a preferential maternal transmission, in keeping with the recent observation that female sex represents a significant positive predictor of fitness. All the parents were identified as affected by 22q11.2DS after the birth of a child with the deletion. All patients, but two, were Caucasian. Demographical features are reported in Additional file 1: Table S1. Clinical data of the second generation subjects were obtained, upon informed consent, through the IPINET Registry or from the referring Units. Data on parents carrying the deletion were collected at each Center. Each subject underwent a clinical and laboratory evaluation protocol (IPINET protocol for 22q11.2DS) available at the site http://www.aieop.org. The protocol included cardiologist examination, echocardiography and abdominal ultrasound, which were performed in order to exclude subclinical cardiac or abdominal defects in asymptomatic subjects. To exclude thyroid and calcium-phosphorus abnormalities, the serum levels of calcium, phosphorus, parathyroid hormone, TSH and FT4 were evaluated. History of speech therapy or speech pathologist interventions were recorded for the evaluation of language disorders. The Wechsler Intelligence Scale for Children (WISC) and the Wechsler Adult Intelligence Scale (WAIS) were used for the assessment of cognitive function in subjects of the second generation and in their parents, respectively. Each Center reported the presence/absence of intellectual disability which was defined as an IQ under 70. Neuropsychiatric evaluation was performed by skilled clinicians using the Schedule For Affective Disorders and Schizophrenia for School-aged Children, Present and Lifetime (K-SADS-PL). The second generation subjects, older than 18 years, and their parents were interviewed with the Structured Clinical Interview for Axis I DSM IV Disorders (SCID).

Among the clinical features, birth defects, facial anomalies, gastrointestinal disorders, infections and autoimmune manifestations were recorded. History of neonatal hypocalcaemia was also considered. In 6 families, data were also obtained from the non-deleted parent, to exclude potential interfering factors not related to the 22q11.2 deletion.

Intrafamilial phenotypic variability was assessed through the evaluation of the clinical phenotype in each parent–child couple. Since in 5 families more than 1 subject with the inherited deletion was detected, the phenotype was analyzed in a total number of 32 parent–child pairs from the 26 families.

### Cytogenetic analysis

The diagnosis of 22q11.2DS was performed by fluorescence *in situ* hybridization (FISH) analysis and/or multiplex ligation-dependent probe amplification (MLPA) using probes for 22q11 region in all affected patients. In one case, the diagnosis was obtained through a CGH array with whole-genome oligonucleotide microarray Agilent Technologies (Santa Clara, CA) according to the manufacturer protocol.

### Statistical analysis

Statistical analysis was performed using the Student”s *t* test or the Fisher exact Test. Values of *P* < 0.05 were considered statistically significant. The calculations were performed using InStat software.

## Results

### Intergenerational clinical phenotypic comparison

Seventeen clinical variables were evaluated in subjects of the second generation and their parents (Table 1), for a total number of 544 and 442 variables in the first and second group, respectively. Overall, affected subjects of the second generation showed a significantly higher number of features than their parents (212 vs 129, *P* = 0.0015). In particular, congenital heart defect (CHD) (62.5 vs 7%, *P* < 0.0001), developmental delay (71.8 vs 42.3%, *P* = 0.032), speech delay (75 vs 46.1%, *P* = 0.031) and calcium-phosphorus abnormalities (37.5 vs 3.8%, *P* = 0.0033) were more represented in the second generation. Conversely, ocular disorders were more frequent in the parents than in their affected children (3.1 vs 23%, *P* = 0.037). Psychiatric (12.5 vs 34.6%), autoimmune (12.5 vs 19.2%) and dental disorders (25 vs 38.4%) tended to be more frequent in the older generation, even though the differences did not reach a statistical significance. No statistically significant difference was observed for the other phenotypic features. About 15% of the clinical features were diagnosed during the study.

**Table 1:** Clinical characteristics of second generation subjects and parents carrying the 22q11.2 deletion

	**Second generation**	**Parents**	
**Total number of subjects**	**32**	**26**	
	**N (%)**	**N (%)**	**p**
Facial anomalies	29 (90.6)	24 (92.3)	1
Congenital heart defect	20 (62.5)	2 (7)	**<0.0001**
Ca-P abnormalities	12 (37.5)	1 (3.8)	**0.0033**
Palatal anomalies	18 (56.2)	13 (50)	0.79
ENT anomalies	4 (12.5)	2 (7)	0.68
Renal disorders	7 (21.8)	2 (7)	0.16
Ocular disorders	1 (3.1)	6 (23)	**0.037**
Neurological disorders	3 (9.3)	1 (3.8)	0.62
Dental anomalies	8 (25)	10 (38.4)	0.39
Skeletal anomalies	15 (46.8)	10 (38.4)	0.6
Gastrointestinal disorders	8 (25)	2 (7)	0.16
Psychiatric disorders	4 (12.5)	9 (34.6)	0.06
Language delay	24 (75)	12 (46.1)	**0.031**
Developmental delay	23 (71.8)	11 (42.3)	**0.032**
Learning difficulty	23 (71.8)	16 (61.5)	0.57
Autoimmunity	4 (12.5)	5 (19.2)	0.71
Infections	9 (28.1)	3 (11.5)	0.19

Only severe infections (sepsis, pneumoniae), requiring hospitalization, or history of recurrent infections were considered. Bold indicate *P* values considered statistically significant.

We next compared the anatomic type of CHD between the 2 groups. We found that in the 20% of the subjects of the second generation with CHD, each individual patient had more than 1 abnormality, whereas in the parent group none of them had a more severe defect. A cyanotic CHD was found only in the 35% of the group with the cardiac defect. In particular, among these patients, we found that 7/20 subjects of the second generation exhibited a tetralogy of Fallot (TOF) and only 1/20 truncus arteriosus (TA). Among those with non cyanotic CHD, a patent ductus arteriosus (PDA) was observed in 4/20, atrial septal defects (ASD) in 4/20, interrupted aortic arch (IAA) in 4/20, ventricular septal defects (VSD) in 3/20, pulmonary valve stenosis (PVS) in 1/20 and other in 2/20. In the parent group, the anomalies found were a PDA in one case and a double aortic arch (DAA) in the other one.

With regard to calcium-phosphorus metabolism abnormalities, 9 subjects in the second generation presented with neonatal hypocalcemia, and in 2 of them a hypoparathyroidism was later diagnosed. Overall, at any age, a total of 5/32 (15.6%) of them received a full diagnosis of hypoparathyroidism. Only 1 subject of the parent group was affected with asymptomatic hypoparathyroidism.

Although the prevalence of the palatal defects was similar in the 2 groups (56 and 50%, respectively), we next compared the type of the defect and made a comparison. Within the group of patients of the second generation, 1 subject had cleft palate and bifid uvula, 16 velopharyngeal insufficiency, 4 hypernasal speech and 4 high arched palate. In the parent group, 11 had velopharyngeal insufficiency, 3 hypernasal speech, 1 high arched palate. Only in two parents, a cleft palate was observed. Thus, no remarkable difference was found for these variables.

Psychiatric disorders were more frequent in the parent group, even though the difference did not reach statistical significance (*P* = 0.06). We found that in the parent group, anxiety was observed in 5/26, mood disorders in 3/26, attention-deficit/hyperactivity disorders in 3/26, behavioral anomalies in 3/26, schizophrenia in 2/26, psychotic disorders not otherwise specified (NOS) in 2/26 and phobia in 1/26. Within the second generation group, 2 of them had behavioral abnormalities represented by trend to social isolation and rejection, impairment in social and daily living skills and low self-esteem, 1 also showed adaptive abnormalities, and the other one an attention deficit disorder. Schizophrenia was observed in 1 subject of the second generation, who, however, was 31 years-old.

Ocular defects were the only anomaly frequently observed in the parental generation, consisting in refractive defects (3/6), strabismus (2/6), retinal vessel abnormalities (1/6), cataract (1/6) and xerophthalmia (1/6). The only child with ocular defect had retinal vessels abnormalities.

### Intrafamilial clinical phenotypic comparison

**Figure 1:**
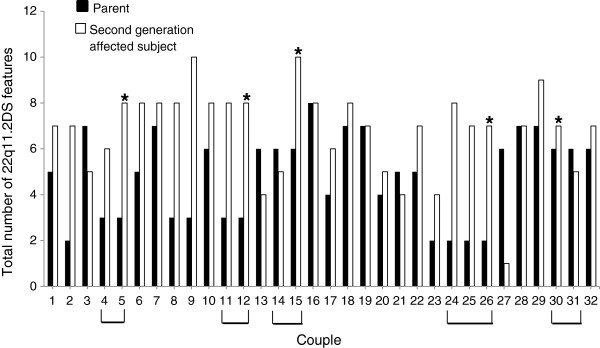
**Intrafamilial phenotypic comparison.** A higher number of features were observed in the second generation in 23 couples. In 6 couples the number of features was higher in the parents’ generation and in the remaining 3 couples no difference was found. The brackets indicate the families with 2 or more second generation affected subjects. * indicates the proband within these families.

With regard to the intrafamilial phenotypic variability, in 23 out of the 32 child/parent couples a higher number of features was found in the second generation, although in 6 couples the number of features was higher in the parents’ generation and in the remaining 3 couples no difference was found (Figure 1). None of the couples with higher number of features in the parents’ generation had a CHD or hypoparathyroidism.

We then performed an intrafamilial evaluation of the clinical severity of the phenotype with regard to the prominent clinical features, whose prevalence was statistically different between the 2 groups, namely CHD, anomalies of the calcium-phosphorus metabolism, developmental and/or speech delay (Table 2).

**Table 2:** Intrafamilial comparison of the clinical severity of the phenotype

**Couple**	**Congenital heart defect**		**Ca-P abnormalities**		**Speech delay**		**Developmental delay**	
	**Affected subject**	**Parent**	**Affected subject**	**Parent**	**Affected subject**	**Parent**	**Affected subject**	**Parent**
**1**	**yes**	**no**	**yes**	**yes**	**no**	**yes**	**yes**	**no**
**2**	**yes**	**no**	**yes**	**no**	**yes**	**no**	**no**	**no**
**3**	**yes**	**no**	**no**	**no**	**no**	**yes**	**yes**	**no**
**°4**	**no**	**no**	**yes**	**no**	**no**	**yes**	**yes**	**no**
**°5**^**δ**^	**yes**	**no**	**yes**	**no**	**yes**	**yes**	**yes**	**no**
**6**	**yes**	**no**	**no**	**no**	**yes**	**yes**	**yes**	**yes**
**7**	**yes**	**no**	**yes**	**no**	**yes**	**no**	**yes**	**no**
**8**	**no**	**no**	**no**	**no**	**yes**	**no**	**yes**	**no**
**9**	**no**	**no**	**no**	**no**	**yes**	**no**	**yes**	**no**
**10**	**no**	**yes**	**yes**	**yes**	**yes**	**yes**	**no**	**no**
**°11**	**yes**	**no**	**yes**	**no**	**no**	**no**	**yes**	**no**
**°12**^**δ**^	**yes**	**no**	**no**	**no**	**yes**	**no**	**yes**	**no**
**13**	**no**	**no**	**no**	**no**	**yes**	**no**	**no**	**yes**
**°14**	**no**	**no**	**no**	**no**	**yes**	**no**	**yes**	**yes**
**°15**^**δ**^	**yes**	**no**	**no**	**no**	**yes**	**no**	**no**	**yes**
**16**	**no**	**no**	**no**	**no**	**yes**	**no**	**yes**	**no**
**17**	**yes**	**no**	**no**	**no**	**yes**	**yes**	**yes**	**yes**
**18**	**yes**	**no**	**no**	**no**	**yes**	**yes**	**yes**	**yes**
**19**	**yes**	**no**	**no**	**no**	**yes**	**yes**	**no**	**yes**
**20**	**yes**	**yes**	**no**	**yes**	**yes**	**yes**	**yes**	**no**
**21**	**yes**	**no**	**yes**	**no**	**yes**	**yes**	**yes**	**yes**
**22**	**yes**	**no**	**yes**	**no**	**yes**	**yes**	**yes**	**yes**
**23**	**yes**	**no**	**yes**	**no**	**yes**	**no**	**yes**	**yes**
**°24**	**no**	**no**	**no**	**no**	**yes**	**no**	**yes**	**no**
**°25**	**no**	**no**	**no**	**no**	**yes**	**no**	**yes**	**no**
**°26**^**δ**^	**yes**	**no**	**yes**	**no**	**yes**	**no**	**yes**	**no**
**27**	**yes**	**no**	**yes**	**no**	**no**	**yes**	**no**	**yes**
**28**	**no**	**no**	**no**	**no**	**yes**	**yes**	**yes**	**yes**
**29**	**no**	**no**	**no**	**no**	**yes**	**no**	**yes**	**no**
**°30**^**δ**^	**yes**	**no**	**no**	**no**	**yes**	**no**	**yes**	**no**
**°31**	**no**	**no**	**no**	**no**	**no**	**no**	**no**	**no**
**32**	**yes**	**no**	**no**	**no**	**no**	**no**	**no**	**no**

°Couple of siblings; ^**δ**^First individual to seek medical attention for genetic evaluation.

In particular, we observed that CHD was present in 18 couples only in the second generation and, in one case, only in the first generation. In the couple in which both members were affected, the parent exhibited a PDA, whereas his child was affected with IAA, thus confirming the lack of phenotypic correspondence. Calcium-phosphorus abnormalities were found in 11 subjects of the second generation, and the only parent with asymptomatic hypoparathyroidism had a child who presented with hypocalcaemia. In 15 couples speech delay was exclusively observed in the second generation whereas in 5 couples only in the first generation. In the remaining 9 couples both members were affected. In 16 couples, developmental delay was exclusively found in the second generation, in 4 cases only in the first generation and in 8 couples both subjects were affected.

To rule out the interference of factors not related to the 22q11.2 deletion, in 6 families, data were collected also from the non-deleted parent. Of note, none of them had a CHD, while 2 children in this subgroup were affected. One subject reported learning and behavioral problems, but not intellectual disabilities, while in another case a borderline IQ of 68 was determined.

Furthermore, in the families with more than one affected child, a milder phenotype was observed in parents than in children, even though the 5 subjects of the second generation, who were diagnosed first, had a higher number of the major clinical features compared to their 6 siblings (16 core features vs 11).

## Discussion and conclusions

We have compared the clinical phenotype of a cohort of 32 subjects affected with inherited 22q11.2DS and their transmitting parents.

In this study, we found a higher number of clinical features and a more severe phenotype in the second generation, which exhibited a higher number of more severe conditions. CHD, abnormalities of the calcium-phosphorus metabolism, developmental and/or speech delay were more represented in children than in parents. It should be considered that, in the past decades, severe CHD was associated with a high neonatal and infant mortality. This evidence was thought to have an appreciable impact on reducing the reproductive fitness of 22q11.2DS patients with CHD. More recently, however, with the improvement of cardiac surgery strategies, it is suggested that a stronger negative selective pressure against the transmission of 22q11.2 deletion is primarily due to the severity of the neuropsychiatric phenotype and intellectual disabilities [27].

The only anomalies more frequent in the parental generation were ocular abnomalities. Since most of them, in particular refractive defects and cataract usually develop during older age, we could not exclude a bias related to the different ages of the subjects in the two groups.

When an intrafamilial comparison of the phenotypic complexity was performed, a higher prevalence of clinical features were found in the second generation. When the comparison concerned the prominent clinical features, whose prevalence was statistically different between the 2 groups, namely CHD, calcium-phosphorus metabolism anomalies, developmental delay and speech delay, we observed that in almost all parent/child couples these major features were more frequent in the second generation. In previous studies [9,10,13-15,24], an intrafamilial variability has already been reported, even though the comparison of intergenerational clinical features has not been performed. An ascertainment bias could partially explain this finding, since the first subject diagnosed within a family is likely to be more severely affected. Moreover, our observation may also be explained by a bias related to the low rate of reproductive fitness of adults with a more severe phenotype.

As to developmental delay, the presence of environmental intellectual disabilities may obviously *per se* influence the mental development of the offspring, because of psychosocial deprivation. However, it should be emphasized that in our cohort the majority of the subjects with developmental delay have a parent without intellectual disability. Moreover, in the 6 families in which also the non-deleted parent was studied, the lack of a clear correlation between environment and the child’ development was noted. As expected, psychiatric disorders were more represented in the first generation, however, the difference was not significant thus suggesting the need of an accurate psychiatric management since childhood.

Several genes, such as *TBX1*[28]*, HIRA, UFD1L*[29] and *CRKL*[30]*,* within the 22q11 region have been considered to be implicated in the pathogenesis of the syndrome. The 22q11.2 phenotype is a developmental field defect, and a DiGeorge-like phenotype may also occur in the absence of the deletion [31], as in diabetic [32] and retinoic acid embryopathy, fetal alcohol syndrome, CHARGE [33] and Fraser syndromes, as well as other chromosomal anomalies, such as 10p13, 17p13, 4q34.1q35.2 [34-36], indicating that several molecules in a common genetic pathway or in functionally related pathways may be involved in 22q11.2DS clinical manifestations.

Several hypotheses have so far been proposed to explain intergenerational and intrafamilial phenotypic variability in genetic syndromes. Deletions of different sizes and location and the extension of an unstable mutation at the 22q11.2 locus could explain the clinical variability [37].

Evidence indicates that *TBX1* gene is sensitive to altered dosage [38], thus leading to the hypothesis that additional alterations of the other allele may explain the clinical variability. In humans, this does not seem to be the case, in that so far DNA variations in *TBX1* locus on the remaining allele were not found in 22q11.2DS patients exhibiting a variable cardiovascular expression and palatal defects. Thus, it is likely that gene modifiers not related to chromosome 22 may be implicated [39,40].

The increased risk of cardiac defects observed in unaffected relatives of 22q11.2DS subjects with CHD, suggested a potential role for genes outside the DiGeorge critical region [41,42]. However, studies aimed at identifying genetic factors outside of the 22q11,2 region, such as *VEGFA*[43] or folate-related genes [44] failed to reveal any association. It should be noted that in the subgroup of families in whom also the non-deleted parent was studied, no CHD was noted suggesting the absence of interfering genetic factors not related to the 22q11.2 deletion in this context.

A copy-number variation may explain a reduced penetrance of some disease-causing mutations [45]. A genetic compensatory effect has also been documented in families of 22q11.2DS subjects, whose clinically normal parent carried 22q11.2 deletion compensated by an insertion of the 22q11.2 critical region inside the other copy of the chromosome [46]. Finally, a mosaic status in the carrier parent, even though rare, could be the explanation of the variable and more benign phenotype. At the moment, we cannot yet exclude the presence of a concomitant duplication or copy number variations in the former generation, since further studies with interphase FISH or array-CGH are required to rule out this hypothesis.

During development, gene expression is accurately orchestrated in time and space in a program that involves enzymes controlling nucleosome remodeling, histone modification and DNA methylation [47]. A demonstration that an epigenetic alteration could result in a DiGeorge syndrome phenocopy has been recently documented in mutant mice lacking the MOZ histone acetyltransferase [48]. Thus, failure of these fine tuned mechanisms could result in an interference of the phenotypic expression.

In our study we also observed that some clinical features were more represented in the previous generation, although the difference did not reach the statistical significance for the limited number of subjects studied. In keeping with recent findings, we observed a higher incidence of psychiatric disorders in the older generation.

The identification of adults with a milder phenotype deserves careful attention since a later onset illness associated with 22q11.2DS has been reported, highlighting the possibility that along with psychiatric disorders, also treatable conditions such as symptomatic or asymptomatic hypocalcaemia, thrombocytopenia and hypothyroidism may occur [49]. Early recognition of these features [50] could provide the benefit of an early treatment [51-53].

### Advantages and limitations of the study

This is the largest cohort of subjects affected with familial 22q11.2DS. A detailed characterization of the clinical features of such subjects, and an intergenerational/intrafamilial clinical comparison has been performed. The observation that within the families, the patients who were first diagnosed had a higher number of core features as compared to their siblings or parents would suggest an ascertainment bias, even though the clinical phenotype of the parents was milder compared to their children. Another possible explanation could be the co-inheritance of a further genetic defect from the non-affected parent. This seems unlikely since this co-inheritance should have occurred in all cases exhibiting the worsening of the phenotype. As for the CHD, it should be noted that none of the non-affected parents had a CHD, thus excluding, at least for this feature, this hypothesis.

## Abbreviations

22q11DS: 22q11.2 Deletion syndrome; LCR: Low-copy repeats; IPINET: Italian network for primary immunodeficiencies; WISC: Wechsler intelligence scale for children; WAIS: Wechsler adult Intelligence Scale; FISH: Fluorescence in situ hybridization; MLPA: Multiplex ligation-dependent probe amplification; CGH: Comparative genoma hybridization; CHD: Congenital heart defect; TOF: Tetralogy of Fallot; TA: Truncus Arteriosus; PDA: Patent Ductus Arteriosus; ASD: Atrial septal defects; IAA: Interrupted Aortic Arch; VSD: Ventricular septal defects; PVS: Pulmonary valve stenosis; DAA: Double aortic arch.

## Competing interests

The authors declare that they have no competing interests.

## Authors’ contributions

CE conceptualized and designed the study, enrolled patients and collected data, drafted the initial manuscript. GG, VG enrolled patients, collected data and coordinated data collection from each group, drafted the initial manuscript. PP, CA, FB, MPC, RC, SM, BM, CM, VM, AP, GS,CC, PR enrolled patients, collected the data, and revised the manuscript. MCD enrolled patients, collected data and critically revised the manuscript. CP conceptualized and designed the study, enrolled patients, collected data and drafted the initial manuscript. MCD and CP equally contributed to the paper. All the authors approved the final version of the manuscript.

## Pre-publication history

The pre-publication history for this paper can be accessed here:

http://www.biomedcentral.com/1471-2350/15/1/prepub

## Supplementary Material

Additional file 1: Table S1 Demographic characteristics of the 22q11.2DS subjects. Click here for file
