# Supplementary material for: Intergenerational and intrafamilial phenotypic variability in 22q11.2 Deletion syndrome subjects
Source: BMC Med Genet. 2014 Jan 2;15:1. doi: 10.1186/1471-2350-15-1 (PMC3893549; doi:10.1186/1471-2350-15-1)
Supplement: Additional file 1: Table S1 — Demographic characteristics of the 22q11.2DS subjects. [file 1471-2350-15-1-S1.doc]

**Additional file 1: Table S1**. Demographic characteristics of the 22q11.2DS subjects

| **Family** | **Affected subject** | **Sex** | **Current age** | **Age at the diagnosis** | **Ethnicity** |
| --- | --- | --- | --- | --- | --- |
| 1 | Parent | M | 38y | 35y | Caucasian |
|  | Subject | F | 12y | 7 d |  |
| 2 | Parent | F | 40y | 33y | Caucasian |
|  | Subject | M | 6y | Prenatal diagnosis |  |
| 3 | Parent | M | 31y | 29y | Caucasian |
|  | Subject | F | 2y | 8m |  |
| 4 | Parent | M | 48y | 44y | Caucasian |
|  | Subject 1 | M | 16y | 6y |  |
|  | Subject 2 | M | 10y | 2m |  |
| 5 | Parent | F | 42y | 33y | Caucasian |
|  | Subject | M | 1y | 7d |  |
| 6 | Parent | F | 36y | 34y | Caucasian |
|  | Subject | M | 14y | 5m |  |
| 7 | Parent | F | 38y | 33y | Caucasian |
|  | Subject | M | 9y | 6y |  |
| 8 | Parent | M | 58y | 40y | Caucasian |
|  | Subject | F | 31y | 18y |  |
| 9 | Parent | F | 42y | 28y | Caucasian |
|  | Subject | F | 14y | 15d |  |
| 10 | Parent | F | 46y | 38y | Caucasian |
|  | Subject 1 | F | 25y | 18y |  |
|  | Subject 2 | M | 13y | 8y |  |
| 11 | Parent | F | 33y | 22y | Caucasian |
|  | Subject | M | 8y | 1m |  |
| 12 | Parent | F | 40y | 37y | Caucasian |
|  | Subject 1 | F | 14y | 4y |  |
|  | Subject 2 | F | 12y | 3y |  |
| 13 | Parent | F | 36y | 34y | Caucasian |
|  | Subject | F | 6y | 4y |  |
| 14 | Parent | M | 21y | 19y | Caucasian |
|  | Subject | F | 2y | 2m |  |
| 15 | Parent | M | **40y** | **39y** | Caucasian |
|  | Subject | M | 9y | 6y |  |
| 16 | Parent | F | **57y** | **50y** | Caucasian |
|  | Subject* | F | 23y | 14y |  |
| 17 | Parent | F | **42y** | **42y** | Caucasian |
|  | Subject | M | 16y | **15y** |  |
| 18 | Parent | F | **33y** | **33y** | Caucasian |
|  | Subject | F | 2y | **1y** |  |
| 19 | Parent | F | 47y | 36y | Indian |
|  | Subject | M | 11y | 2m |  |
| 20 | Parent | F | **39y** | **39y** | Caucasian |
|  | Subject | F | 8y | **8y** |  |
| 21 | Parent | M | **40y** | **40y** | Caucasian |
|  | Subject 1 | M | 9y | **9y** |  |
|  | Subject 2 | F | 8y | **8y** |  |
|  | Subject 3 | F | 3y | **3y** |  |
| 22 | Parent | F | **27y** | **28y** | Japanese |
|  | Subject | F | 4m | 2m |  |
| 23 | Parent | M | **39y** | **41y** | Caucasian |
|  | Subject | F | 8y | **8y** |  |
| 24 | Parent | M | **45y** | **41y** | Caucasian |
|  | Subject | M | 20y | **10y** |  |
| 25 | Parent | F | 37y | 35y | Caucasian |
|  | Subject 1 | M | 13y | 11y |  |
|  | Subject 2 | M | 6y | 5y |  |
| 26 | Parent | M | 41y | 40y | Caucasian |
|  | Subject | M | 2y | 1y |  |

y: years; m:months; d:days ; * subject previously studied in Digilio MC et al, Clin Genet, 2003
